# Impact of eliciting treatment priorities on analgesic prescribing in older patients with high levels of polypharmacy

**DOI:** 10.1093/fampra/cmaf056

**Published:** 2025-07-28

**Authors:** Caroline McCarthy, Barbara Clyne, Susan M Smith, Fiona Boland, Emma Wallace, Michelle Flood, Frank Moriarty

**Affiliations:** Department of General Practice, RCSI University of Medicine and Health Sciences, 123 St Stephen's Green, Dublin D02 YN77, Ireland; Department of Public Health & Epidemiology, School of Population Health, RCSI University of Medicine and Health Sciences, 123 St Stephen's Green, Dublin D02 YN77, Ireland; Discipline of Public Health and Primary Care, Trinity College Dublin, Russell Centre, Tallaght Cross, Dublin D24 DH74, Ireland; Data Science Centre, School of Population Health, RCSI University of Medicine and Health Sciences, 123 St Stephen's Green, Dublin D02 YN77, Ireland; Department of General Practice, University College Cork, Western Gateway, Cork T12 XF62, Ireland; School of Pharmacy and Biomolecular Sciences, RCSI University of Medicine and Health Sciences, 123 St Stephen's Green, Dublin D02 YN77, Ireland; School of Pharmacy and Biomolecular Sciences, RCSI University of Medicine and Health Sciences, 123 St Stephen's Green, Dublin D02 YN77, Ireland

## Abstract

**Background:**

Multimorbidity guidelines recommend tailoring care to patients’ priorities. The Supporting Prescribing in Multimorbidity in Primary Care (SPPiRE) trial focused on optimizing medicines use in older adults with significant polypharmacy and tailoring prescribing and deprescribing to individual priorities. This study aimed to compare self-reported and general practitioner (GP)-recorded patient priorities and examine the impact of prioritizing pain on analgesic prescribing.

**Methods:**

This secondary cohort analysis of the SPPIRE trial and process evaluation assessed baseline participant-identified priorities and intervention group GP-recorded priorities during medication reviews with agreement assessed using Cohen’s kappa. Analgesic prescribing patterns and daily morphine milligram equivalents changes during the study period were summarized. The impact of pain (self-reported, GP-recorded, and severe or extreme pain on the baseline EQ5D) on opioid intensification was analysed using multi-level models accounting for GP practice clustering and intervention effects.

**Results:**

A total of 403 patients (mean age 76.5 years) were included; 178 (44.2%) reported pain as a priority at baseline. Agreement between self-reported and GP-recorded pain was poor (kappa 0.118, *P* = 0.05). Most analgesic prescriptions decreased during the study, except for potent opioids, which increased in both trial arms. All three pain variables were associated with increased odds of opioid intensification at follow-up.

**Conclusion:**

In this older population of patients with significant polypharmacy, identifying pain as a priority was associated with an increased likelihood of opioid intensification, despite guidelines advising against their use for chronic pain. This study highlights the challenges faced by GPs treating pain in older adults with multimorbidity.

**Key messages:**

- Individualized care is important for managing polypharmacy.
- Opioid analgesia is ineffective for chronic pain and there is a dose-dependent risk of harm.
- Self-reported and GP-recorded pain were associated with higher odds of opioid intensification.
- This effect was amplified in those who had a medication review which included eliciting priorities.

## Background

As single-disease models of care are unsuitable for older adults living with multiple conditions, primary care organizations have developed clinical practice guidelines (CPGs) with guiding principles for managing multimorbidity and polypharmacy [1–3]. A central tenet of these CPGs is to ascertain patients’ priorities and individualize treatment. The Supporting Prescribing in Multimorbidity in Primary Care (SPPiRE) intervention followed this approach [4]. The web-guided intervention was delivered by general practitioners (GPs) and targeted patients ≥ 65 years who had high levels of polypharmacy. The intervention was designed to alert GPs to potential inappropriate prescribing, assess individual patient priorities, and tailor prescribing accordingly [5].

The SPPiRE intervention had a significant but small effect on reducing the number of medicines but no evidence of an effect on the overall quality of prescribing [6]. The process evaluation concluded that many patients and clinicians did not engage in the process of eliciting priorities due to deep-seated views around medical decision-making and a preferred paternalistic model by some patients and clinicians [7]. With the priority elicitation process, pain was identified as the most common treatment priority by patients. Treating chronic pain is complex, particularly for older patients with multimorbidity. Recent guidance has advised holistic and individualized assessments with a focus on clear communication, maximizing non-pharmacological treatments and avoiding opioid analgesia [8–11]. However, the prescription of long-term potent opioids continues to rise. In Ireland, from 2011 to 2022, the number of individuals dispensed oxycodone increased by 79%, and 82% of those prescribed a strong opioid received it for at least 4 weeks [9]. Despite recommendations against their use within CPGs, there are multiple barriers to reducing potent opioid use, including perceived pressure from, and desire to do something for, patients [12]. Alternative effective interventions like supporting physical activity and psychological therapies can be difficult to access, particularly within primary care [13].

In this context, the aim of this study was to explore the implementation and effect of eliciting treatment priorities within the SPPiRE intervention, with a focus on pain. Specific objectives were to compare baseline questionnaire self-reported priorities and GP-recorded priorities at medication review and to evaluate changes in analgesic prescribing where pain was reported as a priority.

## Methods

This secondary cohort analysis of the SPPiRE trial and process evaluation used baseline patient questionnaire data, GP-recorded prescriptions, and SPPiRE website usage data, inputted by GPs and is reported in accordance with STROBE guidance for cohort studies, Supplementary Table S1 [14]. Ethical approval was granted from the Irish College of General Practitioners Research Ethics Committee. The primary objective to compare self-reported and GP-recorded priorities was pre-specified prior to conducting the trial and process evaluation. Based on findings from the process evaluation [7], a decision was made to further explore the impact of identifying pain as a priority. This focus was established a priori, as pain was the most commonly identified priority and a priority that could be addressed through prescribing.

### Study population

Eligible practices (those with > 300 older patients), used a finder tool embedded in their software system to identify eligible patients aged ≥ 65 years prescribed ≥ 15 long-term medicines (defined as any unique item with a World Health Organization Anatomical Therapeutic Chemical code on the patient’s current repeat prescription). Recruited GPs invited eligible patients, excluding those unable to give informed consent or receiving palliative care. Between April 2017 and December 2019, 404 patients from 51 general practices were recruited through the Republic of Ireland (403 are included in this analysis due to consent withdrawal). Twenty-six practices (208 patients) were allocated to the intervention group and 25 practices were randomly allocated to the control group. Intervention GPs were given access to online educational material and performed a once-off GP-guided, deprescribing-orientated medication review. Control practices delivered usual care. In total, 163 of the 208 intervention patients had a medication review, and practices were given 6 months to complete all their reviews.

### Data collection

Patient demographics and patient-reported outcomes were collected via baseline postal questionnaires. Participants’ priorities were gathered by asking: ‘We are interested in finding out about your priorities for treatment. For example, your priorities may be treating pain, treating other symptoms (e.g. breathlessness), or maintaining independence. Please write down your health priority/priorities in the box below’. Prescription data were collected directly from practices at follow-up for both time points. During the study period, 21 participants died and 15 moved practice, for these participants the baseline prescription was carried forward. The development of the SPPiRE intervention has been described elsewhere [15]. Briefly, intervention GPs had access to training videos where concepts such as potentially inappropriate prescribing, treatment burden, and individualized care were introduced. During the priorities component of the medication review, GPs were prompted to ask the patient about their overall treatment priorities and to record these data on the SPPiRE website.

### Variables of interest

Patient priorities were identified from baseline questionnaires and coded into predefined categories by the lead author (C.M.C.) using a coding framework based on a health outcome prioritization tool previously developed for older patients with multimorbidity undergoing medication review [16, 17]. Categories included: pain (treating pain/a painful condition), other symptoms (e.g. fatigue), staying alive (aims to live as long as possible), and maintaining independence (mobility and self-sufficiency). During the coding process, three other categories were developed, mood (concerns about stress/low mood), treating a specific condition (e.g. managing diabetes), and reducing treatment burden (desire to stop medications/reduce appointments). Some patients did not record any priorities. During the intervention medication review with patients, GPs were prompted to record patients’ treatment priorities. Medication reviews took place on average 9 months after baseline data collection [median 283 days from baseline (IQR 200-425)]. Free-text priority data recorded by GPs were coded in the same way as patient questionnaire priorities by the same author (C.M.C.).

Prescribing data included strength, dose, and total quantity, but did not capture instructions such as “as needed” use. Analgesic prescriptions were classified into potent opioids, weak opioids, systemic non-steroidal anti-inflammatory drugs (NSAIDs), topical NSAIDs, gabapentinoids, amitriptyline, and paracetamol (Supplementary Text S1). For weak and potent opioids, daily morphine milligram equivalents (MMEs) were calculated to facilitate comparison across different opioid medications (Supplementary Text S1). Total MME for each participant was calculated at baseline and follow-up. Follow-up was 6 months after the medication review. For control patients, follow-up was 6 months after the medication review in the matched intervention practice (which was either allocated at the same time or at the nearest corresponding time point). The median number of days from baseline to follow-up was 474 (IQR 384-564). The difference between MME at baseline and follow-up identified participants with opioid intensification during the study.

### Analysis

Patient demographics, reported priorities, and changes in analgesic prescribing during the trial were summarized descriptively. Cohen’s kappa was used to assess agreement between self-reported and GP-recorded priorities for patients who had an SPPiRE medication review. The potential impact of self-reported pain as a treatment priority on opioid intensification was explored using multi-level modelling, accounting for clustering by GP, and any intervention effect. Given that the intervention was a deprescribing-orientated medication review, an interaction between self-reported priorities and the intervention was also included, and the predicted probabilities with 95% confidence intervals were calculated and graphed to illustrate the interaction effect on opioid intensification. For comparison, the impact of an alternative explanatory variable, severe, or extreme pain on the EQ5D-5L [18] pain domain from the baseline questionnaire, on opioid intensification was also explored, accounting for clustering and any intervention effect in the same manner. The effect of GP-recorded pain as a treatment priority during the medication review on opioid intensification was similarly explored using multi-level modelling among intervention group patients, adjusting for clustering by GP. Analyses were performed in Stata version 18 [19], with both adjusted and unadjusted estimates presented for all models. Adjusted models accounted for age, gender, and baseline number of medications as potential confounders. Potential confounders were identified from variables collected in the trial through clinical judgement and consensus among the research team, based on their likely association with opioid prescribing in routine practice.

## Results

Among the 403 included patients (57% female, mean age 76.4 years, SD 6.8), the mean number of medicines at baseline was 17.5 (SD 3.6), see Supplementary Table S2 for a summary of baseline characteristics. There was a median of one self-reported priority recorded on baseline questionnaires (IQR 1-2) with 80 participants (19.8%) not recording any. Of the 208 intervention group participants, 163 attended a SPPiRE medication review. Of these, there was also a median of one GP-recorded priority (IQR 1-2) with no priority recorded for 38 participants (23.3%). There was generally poor agreement between self-reported and GP-recorded priorities across all domains (Table 1). Statistically significant kappa scores were observed in the domains of pain, mood, and treatment of specific symptoms, suggesting stronger evidence of poor agreement in these areas. However, the magnitude of kappa scores in these domains was similar to those in other categories.

**Table 1.** Comparison of self-reported and GP-recorded priorities.

	All participants (*n* = 403)	Participants who had a SPPiRE review (*n* = 163)				
Priority type	Self-report *N* (%)	Self-report *N* (%)	GP report *N* (%)	Observed agreement (%)	Kappa	*P*-value
Pain	178 (44.2)	79 (48.5)	50 (30.7)	56.4	0.118	0.05
Other symptoms	133 (33.0)	55 (33.7)	63 (38.4)	52.2	−0.033	0.67
Maintain independence	123 (30.5)	48 (29.5)	21 (12.9)	67.5	0.069	0.18
Treat specific condition	82 (20.4)	31 (19.0)	19 (11.7)	77.9	0.158	0.02
None recorded	73 (18.1)	29 (17.8)	38 (23.3)	69.9	0.077	0.14
Mood	18 (4.5)	9 (5.5)	12 (7.4)	90.8	0.238	0.01
Reduce treatment burden	7 (1.7)	2 (1.2)	17 (10.4)	88.3	−0.022	0.69
Staying alive	1 (0.3)	0 (0)	3 (1.8)	98.2	0.000	0.50

At baseline, across all participants, 7036 medicines were prescribed, including 733 (10.1%) analgesics, reducing to 6763 medicines and 701 (10.4%) analgesics at follow-up. At the patient level, 332 patients (82.4%) were prescribed at least one analgesic, reducing to 324 (80.4%) at follow-up. Prescriptions decreased for weak opioids (−26 prescriptions, −20%), systemic NSAIDs (−18, −34.7%), gabapentinoids (−9, −8.1%), and amitriptyline (−3, −8.3%). Prescriptions increased for potent opioids (+ 18, + 21.2%) and paracetamol (+ 6, + 3.1%), while topical NSAID prescriptions remained unchanged. Prevalence of potent opioid prescribing increased from 76 patients (18.9%) at baseline to 81 (20.1%) at follow-up. The mean MME per patient remained similar 14.33–14.01 mg (mean difference −0.27, *P* = 0.84). Supplementary Table S3a–c for details on prescriptions by analgesic type, opioid intensification, MME, time-point, and self-reported priority of pain.

Table 2 shows changes in analgesia prescribing based on pain identified as a priority at baseline and during the SPPIRE medication review.

**Table 2.** Analgesic medication changes by GP-recorded and self-reported priorities.

Analgesia change^a^	Baseline self-reported pain (*n* = 403)		
	No, *n* = 225 (%)	Yes, *n* = 178 (%)	Adjusted odds ratio (95%CI)^c^
New analgesic added^b^	69 (30.7)	71 (39.9)	1.53 (0.98–2.33)
Analgesic discontinued	83 (36.9)	74 (41.6)	1.07 (0.71–1.63)
Analgesic dose change	103 (45.8)	112 (69.9)	1.15 (0.53–2.50)
No change to analgesia	67 (29.78)	25 (14.0)	0.41 (0.24–0.70)
Opioid Intensification	23 (10.2)	31 (17.4)	1.80 (1.01–3.22)
Analgesia change^a^	GP-recorded pain (*n* = 163) at medication review		
	No, *n* = 113 (%)	Yes, *n* = 50 (%)	Adjusted odds ratio (95%CI)^c^
New analgesic added^b^	34 (30.1)	20 (40.0)	1.47 (0.70–3.08)
Analgesic discontinued	43 (38.1)	24 (48.0)	1.47 (0.75–2.89)
Analgesic dose change	56 (49.6)	31 (62.0)	1.99 (0.93–2.98)
No change to analgesia	36 (31.9)	3 (6.0)	0.12 (0.33–0.48)
Opioid intensification	17 (15.0)	13 (26.0)	2.73 (1.21–6.17)

^a^Patients may have had multiple medication changes, thus the sum of changes is > 100%.

^b^Any prescription for an opioid, systemic or topical NSAID, gabapentinoid, paracetamol, or amitriptyline present at follow-up that was not prescribed at baseline.

^c^Multilevel model that accounted for clustering within GP practices, age, sex, number of medicines, and potential intervention effects in models including both trial arms.

**Figure 1.**
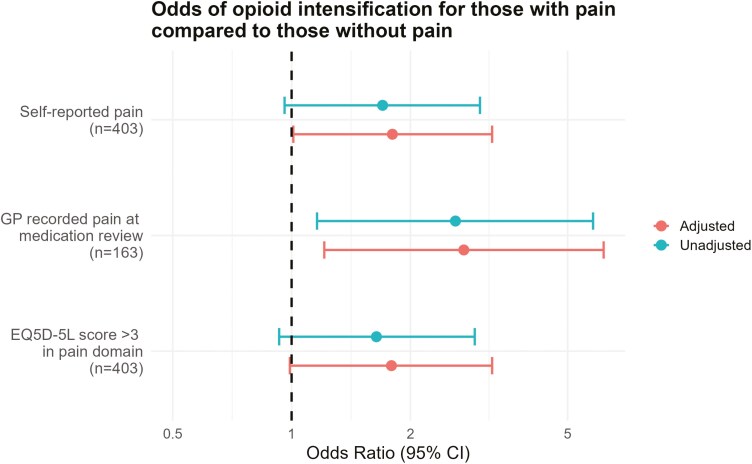
Multi-level logistic regression exploring the effect of self-reported pain, GP-recorded pain, or severe or extreme pain on the EQ5D-5L (compared to no pain) on opioid intensification. Models including both trial arms (*n* = 403) account for any potential effect of the intervention. All models adjust for clustering within GP practices, while adjusted models additionally control for age, gender, and the number of medicines at baseline.

Participants self-reporting pain as a priority at baseline had higher odds of opioid intensification during the study period (OR: 1.80, 95%CI: 1.01–3.22, *P* = 0.05). A similar effect was observed for GP-recorded pain as a priority among intervention participants with a medication review (OR: 2.73, 95%CI: 1.21–6.17, *P* = 0.02). Similarly, for severe or extreme pain on the EQ5D-5L at baseline for all participants, there was a potential association with opioid intensification (OR: 1.79, 95%CI: 0.99–3.22, *P* = 0.05), although this did not reach the conventional threshold for statistical significance Figure 1 and Supplementary Table S4.

**Figure 2.**
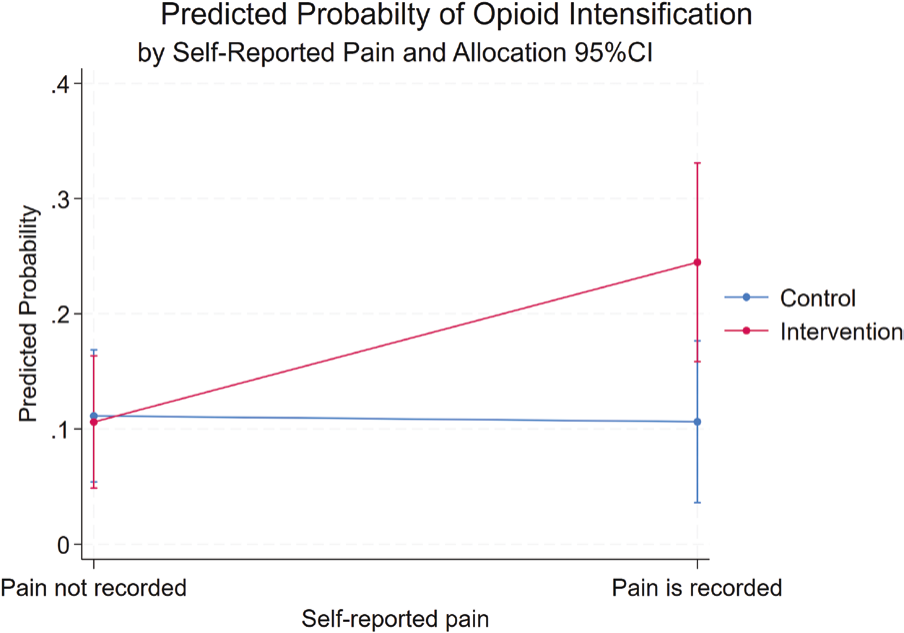
Marginal predicted probability of opioid intensification by pain and allocation group. The reference group is patients in the control group with no self-reported pain. Predictions are adjusted for clustering within GP practices, age, gender, and number of medications. Error bars represent 95% confidence intervals.

The effect of self-reported pain on opioid intensification appeared larger in the intervention group compared to the control group (OR: 2.89, 95%CI: 086–9.72, *P* = 0.09). The predicted probability for opioid intensification among those who self-reported pain as a priority in the intervention group was 0.24 (95%CI: 0.16–0.33) and in the control group was 0.11 (95%CI: 0.04–0.18), however, the difference was not statistically significant, Figure 2.

## Discussion

### Summary of results

Pain was identified as a priority in 44% of these older adults with high levels of polypharmacy and multimorbidity. Self-reported and GP-recorded treatment priorities showed poor agreement, consistent with findings from a systematic review of studies exploring treatment prioritization in patients with multimorbidity [20]. Pain and other symptoms were identified more commonly in this study, potentially reflecting the high morbidity and treatment burden of participants [20]. Twenty percent of patients did not identify a priority, and GPs did not record one for 23% of patients during medication reviews. This could indicate that some participants were satisfied with their current state [21], or it might highlight the practical challenges of addressing priorities, as evidence suggests that patients’ existential concerns about enduring illness, ageing, and mortality often remain unresolved [22].

Consistent with national trends, potent opioid prescriptions increased, however, unlike national trends the MME per person remained similar and there was a decrease in prescriptions for weak opioids and gabapentinoids [23]. Unlike national data, SPPiRE data represents prescribed rather than dispensed medicines complicating comparisons, particularly for medicines prescribed on an as-needed basis. All three pain variables included in this analysis appeared to increase the odds of opioid intensification at follow-up.

Of note, there were no specific national policies introduced during the study period to address rising levels of opioid prescribing. More recently, there has been updated guidance recommending their avoidance for chronic non-cancer pain and limiting their duration for treating acute non-cancer pain [9, 24]. This is in line with international CPGs that have been published recently and recommend avoiding opioids for chronic pain [10, 25].

### Strengths and limitations

A major strength of this work is the triangulation of multiple data sources to address a novel question among this subset of patients with very high levels of polypharmacy. In addition, calculating the daily patient-level MME is an approach that allows for meaningful comparison within and between individuals. An important limitation of this analysis is the time between priorities being self-reported on baseline questionnaires and intervention GPs recording priorities during the SPPiRE review. These patients with very significant disease and treatment burdens may have identified different priorities in their medication review. Secondly, it is possible that GPs’ prescribing patterns were influenced by other components of the intervention, for example approximately half of NSAID prescriptions at baseline were identified as potentially inappropriate [26] (Supplementary Table S5). Finally, these data reflect prescribed doses, not necessarily what was dispensed which may particularly affect medicines prescribed on an as-needed basis.

### Implications for practice, policy, and future research

These patients present a high level of complexity, both in terms of their health conditions and interactions with healthcare providers. The discrepancy between GP-recorded and self-reported priorities underscores the challenge of operationalizing an apparently simple recommendation in clinical guidelines for multimorbidity, which requires complex behaviours in clinicians and patients around effective communication. It also raises important questions about the potential effect of identifying patient priorities without access to evidence-based, effective interventions. This approach may not improve outcomes if targeted interventions to support clinicians in addressing these priorities are lacking and risks reinforcing suboptimal care by identifying issues that cannot be adequately addressed within the current system. Physical activity and psychological therapies have been identified as safer, more effective approaches to treating chronic pain [11]. However, clinicians have identified multiple barriers to adhering to these guidelines including diagnostic ambiguity and limited access to non-pharmacological treatments [27]. Multi-disciplinary care is important, particularly for older patients with multimorbidity, and may help reduce iatrogenic harm from unnecessary medicines use. There is evidence that direct access to physiotherapy may be associated with less medication use and lower healthcare costs [28] and the use of pharmacists in general practice are also cost-effective and associated with improved prescribing [29]. Also, structured multi-disciplinary geriatric assessments may reduce mortality and morbidity and enhance daily functioning in older adults [30], and it is likely that very complex patients may be most likely to benefit.

There is some evidence that chronic pain CPGs may be associated with reduced opioid prescribing [31]. Given that many of these guidelines have been published within the past 5 years, their full impact on prescribing behaviour may not yet be apparent [11]. However, efforts should be made to estimate their effect, for example through quasi-experimental designs. Enhanced clinician knowledge, through both medical education and updated clinical guidelines, is essential to support shared decision-making, enabling prescribers to clearly communicate the risks of these medicines and their limited effectiveness in managing chronic pain. However, given that doctors’ ‘desire to do something’ can be a barrier to avoiding low-value care such as opioid prescribing for chronic pain [12], improving access to effective non-pharmacological alternatives such as physiotherapy will be important. In addition to guidance, broader strategies such as audit and feedback mechanisms and regulatory controls are other approaches to address rising opioid prescribing. Finally, the role of structural and commercial factors should not be overlooked. The US opioid crisis was in large part fuelled by aggressive marketing from pharmaceutical companies. This has led to renewed calls for stronger separation between pharmaceutical interests and clinical research, education, and guideline development to safeguard public health [32].

## Conclusion

Eliciting treatment priorities and tailoring care for older adults with multimorbidity is complex and may be difficult to operationalize and measure in primary care, with current evidence suggesting poor agreement between clinicians and patients. Chronic pain was identified as the most common treatment priority in this population of older adults with high levels of polypharmacy. The identification of pain as a treatment priority during medication review was associated with higher odds of opioid intensification despite guidance advising against their use for chronic pain. However, limited effective and safe therapeutic interventions and access to non-pharmacological treatments are barriers to optimal chronic pain management. Older patients with high disease and treatment burden levels require access to multi-disciplinary care, with support from professionals such as pharmacists, physiotherapists, and geriatricians to address their complex needs holistically and reduce the risk of medication-related harm.

## Supplementary Material

cmaf056_suppl_Supplementary_Tables_1-S5_Texts_S1

## Data Availability

The minimized trial dataset is available here: https://zenodo.org/records/5539817. The minimized prescription dataset used in this study is available here: https://zenodo.org/records/6594742. However, these datasets are not linked to avoid potential identifiability. The dataset for this study was created by triangulating multiple sources and the minimized dataset is available from the corresponding author upon reasonable request. The Stata do file with the code used to perform the analyses described in this study is available here: https://zenodo.org/records/15361049.
