# Supplementary material for: Impact of eliciting treatment priorities on analgesic prescribing in older patients with high levels of polypharmacy
Source: Fam Pract. 2025 Jul 28;42(4):cmaf056. doi: 10.1093/fampra/cmaf056 (PMC12302712; doi:10.1093/fampra/cmaf056)
Supplement: cmaf056_suppl_Supplementary_Tables_1-S5_Texts_S1 [file cmaf056_suppl_supplementary_tables_1-s5_texts_s1.pdf]

Supplemental Table S1: STROBE Checklist of items that should be included in reports of cohort studies

	Item No	Recommendation	Location
**Title and abstract**	1	(*a*) Indicate the study's design with a commonly used term in the title or the abstract	Abstract
		(*b*) Provide in the abstract an informative and balanced summary of what was done and what was found	Abstract
**Introduction**			
Background/rationale	2	Explain the scientific background and rationale for the investigation being reported	Background
Objectives	3	State specific objectives, including any prespecified hypotheses	Background
**Methods**			
Study design	4	Present key elements of study design early in the paper	Methods
Setting	5	Describe the setting, locations, and relevant dates, including periods of recruitment, exposure, follow-up, and data collection	Methods, study population
Participants	6	(*a*) Give the eligibility criteria, and the sources and methods of selection of participants. Describe methods of follow-up	Methods, study population
		(*b*) For matched studies, give matching criteria and number of exposed and unexposed	N/A
Variables	7	Clearly define all outcomes, exposures, predictors, potential confounders, and effect modifiers. Give diagnostic criteria, if applicable	Methods, Variables of interest
Data sources/ measurement	8*	For each variable of interest, give sources of data and details of methods of assessment (measurement). Describe comparability of assessment methods if there is more than one group	Methods, Variables of interest and Data collection
Bias	9	Describe any efforts to address potential sources of bias	Methods, Analysis

Study size	10	Explain how the study size was arrived at	Methods, study population
Quantitative variables	11	Explain how quantitative variables were handled in the analyses. If applicable, describe which groupings were chosen and why	Methods, Analysis
Statistical methods	12	(*a*) Describe all statistical methods, including those used to control for confounding	Methods, Analysis
		(*b*) Describe any methods used to examine subgroups and interactions	Methods, Analysis
		(*c*) Explain how missing data were addressed	Methods, Data collection
		(*d*) If applicable, explain how loss to follow-up was addressed	Methods, Data collection
		(*e*) Describe any sensitivity analyses	N/A
**Results**			
Participants	13*	(a) Report numbers of individuals at each stage of study—eg numbers potentially eligible, examined for eligibility, confirmed eligible, included in the study, completing follow-up, and analysed	Methods, study population (secondary analysis of data already collected)
		(b) Give reasons for non-participation at each stage	Methods, study population
		(c) Consider use of a flow diagram	
Descriptive data	14*	(a) Give characteristics of study participants (eg demographic, clinical, social) and information on exposures and potential confounders	Line 1 Results, Supplemental Table 2
		(b) Indicate number of participants with missing data for each variable of interest	Methods, study population

		(c) Summarise follow-up time (eg, average and total amount)	Methods, Variables of interest
Outcome data	15*	Report numbers of outcome events or summary measures over time	Supplemental S3 Tables
Main results	16	(*a*) Give unadjusted estimates and, if applicable, confounder-adjusted estimates and their precision (eg, 95% confidence interval). Make clear which confounders were adjusted for and why they were included	Figure 1, Supplemental Table S4
		(*b*) Report category boundaries when continuous variables were categorized	N/A
		(*c*) If relevant, consider translating estimates of relative risk into absolute risk for a meaningful time period	N/A
Other analyses	17	Report other analyses done—eg analyses of subgroups and interactions, and sensitivity analyses	N/A
**Discussion**			
Key results	18	Summarise key results with reference to study objectives	Discussion, Summary of results
Limitations	19	Discuss limitations of the study, taking into account sources of potential bias or imprecision. Discuss both direction and magnitude of any potential bias	Discussion, Strengths and limitations
Interpretation	20	Give a cautious overall interpretation of results considering objectives, limitations, multiplicity of analyses, results from similar studies, and other relevant evidence	Discussion, Summary of results
Generalisability	21	Discuss the generalisability (external validity) of the study results	Discussion, Summary of results, Implications for practice
**Other information**			
Funding	22	Give the source of funding and the role of the funders for the present study and, if applicable, for the original study on which the present article is based	Funding information

Supplemental Table S2: Baseline characterises by self-reported pain status

Characteristic	Pain is not self-reported as priority on baseline questionnaire (n=225)	Pain is self-reported as priority on baseline questionnaire (n=178)
Mean age (SD)	76.4 (6.6)	76.6 (6.8)
Mean baseline number of medicines (SD)	17.0 (3.4)	18.0 (3.7)
Female sex (%)	119 (52.9)	112 (62.9)
EQ5D-5L		
• No pain	34 (16.2)	0 (0)
• Mild pain	56 (26.7)	25 (15.0)
• Moderate pain	84 (40.0)	62 (37)
• Severe pain	30 (14.3)	65 (39)
• Extreme pain	6 (2.9)	15 (9.0)
Mean MME at baseline (SD)	10.4 (36.5)	19.3 (43.2)

Supplemental Text S1: Development of Analgesic Variables and MME

Analgesic Categories

1. **Weak Opioids**
    - Weak opioids were identified using the following ATC codes:
        - **N02AX02** and **N02AX52**
        - Combination agents under **N02AJ**
2. **Potent Opioids**
    - Potent opioids were defined as any drugs with ATC codes starting with **N02A**, excluding:
        - Combination agents under **N02AJ**
        - Weak opioids identified with **N02AX02** or **N02AX52**
3. **Systemic NSAIDs**
    - Systemic non-steroidal anti-inflammatory drugs (NSAIDs) were identified using ATC codes starting with **M01A** excluding glucosamine.
4. **Gabapentinoids**
    - Gabapentinoids were identified using the following ATC codes:
        - **N03AX12** for gabapentin
        - **N03AX16** for pregabalin
5. **Amitriptyline**
    - Amitriptyline was identified using the ATC code **N06AA09**. All prescribing was assumed to be for pain.
6. **Paracetamol**
    - Paracetamol was identified using the ATC code **N02BE01**.
7. **Topical NSAIDs**
    - Topical NSAIDs were identified using ATC codes starting with **M02AA**.

Daily dose calculation

The mean daily dose for each analgesic was calculated in milligrams using strength and quantity variables.

Calculation of Morphine Milligram Equivalent (MME) Daily Dose (1)

1. Morphine MME = Daily dose x 1.0
2. Oxycodone MME = Daily dose × 1.5
3. Fentanyl patches MME = mcg/hr x 2.7
4. Buprenorphine MME = mcg/hr x 2.2
5. Tapentadol MME=Daily dosage × 0.4
6. Codeine/ tramadol MME = Daily dosage x 0.1

Reference

1. Nielsen S, Degenhardt L, Hoban B, et al. A synthesis of oral morphine equivalents (OME) for opioid utilisation studies. 2016;25(6):733-7.

Supplemental Tables S3a, b and c: Changes in analgesia prescribing by time-point and self-reported pain

S3a: Distribution of pain medications by self-reported pain at baseline and follow-up

Medication Type	Pain reported as priority N=178		Pain not reported as priority N=225	
Time point	Baseline N (%)	Follow-up N (%)	Baseline N (%)	Follow-up N (%)
Systemic NSAID	27 (15.2)	16 (9.0)	23 (10.2)	18 (8.0)
Potent Opioid	48 (27.0)	51 (28.7)	28 (12.4)	30 (13.3)
Weak Opioid	69 (38.8)	57 (32.0)	49 (21.8)	43 (19.1)
Gabapentinoid	63 (35.4)	58 (32.6)	45 (20.0)	41 (18.2)
Amitriptyline	23 (12.9)	22 (12.4)	13 (5.8)	11 (4.9)
Paracetamol	87 (48.9)	95 (53.4)	108 (48.0)	106 (47.1)
Topical NSAID	59 (33.2)	59 (33.2)	61 (27.1)	59 (26.2)

S3b: Mean milligram morphine equivalent by self-reported pain at baseline and follow-up

MME	Pain reported as priority N=178		Pain not reported as priority N=225	
Time point	Baseline Mean (SD)	Follow-up Mean (SD)	Baseline Mean (SD)	Follow-up Mean (SD)
MME	19.30 (43.23)	19.16 (44.62)	10.40 (36.55)	10.04 (35.45)

S3c: Opioid intensification and change in mean milligram morphine equivalent at follow-up by self-reported pain

	Pain reported as priority N=178	Pain not reported as priority N=225
Opioids intensified N (%)	32 (18.0)	25 (11.1)
Mean change in MME	+31.6mg	+19.76mg

Supplemental Table S4: Multilevel logistic regression exploring the effect of baseline reported pain on opioid intensification

Explanatory variable	Unadjusted OR (95% CI)	Adjusted* OR (95% CI)
Self-reported pain (n=403)$^{\mu}$	1.70 (0.96-3.00)	1.80 (1.01-3.32)
Self-reported pain with intervention allocation (n=403)^	2.92 (0.87-9.78)	2.89 (0.86-9.72)
Severe or extreme pain on the EQ5D (n=403) $^{\mu}$	1.64 (0.93-2.91)	1.79 (0.99-3.22)
GP recorded pain at medication review (n=163)	2.60 (1.16-5.80)	2.73 (1.21-6.17)

**Adjusted for age, gender and number of medicines at baseline. All models adjusted for the effect of clustering.*

$^{\mu}$ *Adjusted for allocation*

^ Interaction term between self-reported and allocation

Supplemental Table S5: Analgesic prescribing by allocation, time-point and PIP

Drug group	Intervention				Control			
	Baseline N	PIP N (%)	Follow-up N	PIP N (%)	Baseline N	PIP N (%)	Follow-up N	PIP N (%)
Systemic NSAID	29	8 (27.6)	18	2 (11.1)	21	7 (33.3)	16	0 (0)
Potent Opioid	36	8 (22.2)	40	11 (27.5)	40	6 (15)	41	7 (17.1)
Weak Opioid	61	16 (26.2)	57	7 (12.3)	57	15 (26.3)	43	5 (11.6)
Gabapentinoid	51	13 (25.5)	48	5 (10.4)	57	12 (21.1)	51	6 (11.8)
Amitriptyline	16	5 (31.3)	13	3 (23.1)	20	3 (15.0)	20	3 (15.0)
Paracetamol	95	19 (20)	94	17 (18.1)	100	14 (14.0)	107	12 (11.2)
Topical NSAID	69	12 (17.4)	62	13 (21.0)	51	11 (21.6)	56	5 (8.9)
